# EUS-guided tissue acquisition in the study of the adrenal glands: Results of a nationwide multicenter study

**DOI:** 10.1371/journal.pone.0216658

**Published:** 2019-06-06

**Authors:** A. Martin-Cardona, G. Fernandez-Esparrach, J. C. Subtil, J. Iglesias-Garcia, M. Garcia-Guix, A. Barturen Barroso, A. Z. Gimeno-Garcia, J. M. Esteban, A. Pardo Balteiro, A. Velasco-Guardado, E. Vazquez-Sequeiros, C. Loras, B. Martinez-Moreno, A. Castellot, C. Huertas, M. Martinez-Lapiedra, A. Sanchez-Yague, A. Teran, V. J. Morales-Alvarado, M. Betes, D. de la Iglesia, C. Sánchez-Montes, M. D. Lozano, J. Lariño-Noia, A. Gines, C. Tebe, J. B. Gornals, On belhaf of Spanish Group for EUS-Guided TA in the adrenal gland

**Affiliations:** 1 Endoscopy Unit, Department of Digestive Diseases, Hospital Universitari de Bellvitge, Bellvitge Biomedical Research Institute (IDIBELL), University of Barcelona, Barcelona, Spain; 2 Department of Digestive Diseases, Hospital Universitari Mútua Terrassa, Fundació per la Recerca Mútua Terrassa, CIBERehd, Terrassa, Spain; 3 Endoscopy Unit, ICMDiM, Hospital Clinic, IDIBAPS, CIBEREHD, University of Barcelona, Barcelona, Spain; 4 Endoscopy Unit, University of Navarra Clinic, Pamplona, Spain; 5 Department of Gastroenterology and Hepatology, University Hospital of Santiago de Compostela, Santiago, Spain; 6 Department of Digestive Diseases, Hospital Universitario Cruces, Bilbao, Spain; 7 Department of Gastroenterology and Hepatology, Hospital Universitario de Canarias, Tenerife, Spain; 8 Endoscopy Unit, Department of Digestive Diseases, Hospital Clínico San Carlos, Madrid, Spain; 9 Department of Digestive Diseases, Hospital Universitario Joan XXIII, Tarragona, Spain; 10 Department of Digestive Diseases, Hospital Universitario de Salamanca, Instituto de Investigación Biomédica de Salamanca (IBSAL), Salamanca, Spain; 11 Endoscopy unit, Gastroenterology and Hepatology Service, Hospital Ramon y Cajal, IRYCIS, Madrid, Spain; 12 Health Sciences, Universitat Oberta de Catalunya, Barcelona, Spain; 13 Department of Digestive Diseases, Hospital General Universitario de Alicante, Alicante, Spain; 14 Department of Digestive Diseases, Hospital Insular de Gran Canaria, Las Palmas de Gran Canaria, Spain; 15 Department of Digestive Diseases, Hospital Dr. Josep Trueta Girona, Girona, Spain; 16 Instituto Oncológico Valenciano, Valencia, Spain; 17 Endoscopy Unit, Hospital Costa Sol, Marbella, Spain; 18 Department of Digestive Diseases, Hospital Universitario Marqués de Valdecilla, Santander, Spain; 19 Biostatistics Unit, Institute of Biomedical Research of Bellvitge, L’Hospitalet de Llobregat, Barcelona, Spain; Medical University of Vienna, AUSTRIA

## Abstract

**Background:**

There are limited data about the role of endoscopic ultrasound-guided tissue acquisition (EUS-TA), by fine needle aspiration (EUS-FNA) or biopsy (EUS-FNB), in the evaluation of the adrenal glands (AG). The primary aim was to assess the diagnostic yield and safety. The secondary aims were the malignancy predictors, and to create a predictive model of malignancy.

**Methods:**

This was a retrospective nationwide study involving all Spanish hospitals experienced in EUS-TA of AGs. Inclusion period was from April-2003 to April-2016. Inclusion criteria: all consecutive cases that underwent EUS-TA of AGs. EUS and cytopathology findings were evaluated. Statistical analyses: diagnostic accuracy of echoendoscopist’s suspicion using cytology by EUS-TA, as gold standard; multivariate logistic regression model to predict tumor malignancy.

**Results:**

A total of 204 EUS-TA of AGs were evaluated. Primary tumor locations were lung70%, others19%, and unknown11%. AG samples were adequate for cytological diagnosis in 91%, and confirmed malignancy in 60%. Diagnostic accuracy of the endosonographer's suspicion was 68%.

The most common technique was: a 22-G (65%) and cytological needle (75%) with suction-syringe (66%). No serious adverse events were described. The variables most associated with malignancy were size>30mm (OR2.27; 95%CI, 1.16–4.05), heterogeneous echo-pattern (OR2.11; 95%CI, 1.1–3.9), variegated AG shape (OR2.46; 95%CI, 1–6.24), and endosonographer suspicion (OR17.46; 95%CI, 6.2–58.5). The best variables for a predictive multivariate logistic model of malignancy were age, sex, echo-pattern, and AG-shape.

**Conclusions:**

EUS-TA of the AGs is a safe, minimally invasive procedure, allowing an excellent diagnostic yield. These results suggest the possibility of developing a pre-EUS procedure predictive malignancy model.

## Introduction

The adrenal gland (AG) is a frequent location where metastatic cells settle in patients with known primary tumor, mainly of the lung, involving a poor prognosis and a change in management. Previous data show that the incidence of AG masses in patients with lung cancer ranges from 4% to 18%, of which 40% are malignant [1,2]. However, up to two-thirds of adrenal masses, in lung cancer, are benign adenomas [3]. The data provided by imaging techniques cannot accurately differentiate between a benign and a malignant mass in clinical practice, and the need to obtain material is common [4–6].

The available data have shown that percutaneous image-guided tissue acquisition (TA) of the AG has a high sensitivity and accuracy in diagnosing AG lesions. However this technique yields nondiagnostic samples in up to 19% of cases and is not exempt from adverse events (AE), such as needle tract metastasis, hemorrhage, and pneumothorax [7–10].

On the other hand, endoscopic ultrasound-guided TA, by fine needle aspiration (EUS-FNA) or fine needle biopsy (EUS-FNB) has proven to be very effective in obtaining samples of various targets (lymph nodes, subepithelial tumours, and pancreas). To date, however, there are few studies published showing that EUS-FNA in the study of AG is a minimally invasive technique with less AE compared with percutaneous guided punctures [3, 10–13]. It is reported that EUS-FNA of AGs is sensitive and useful to differentiate between malignant and benign masses in cases of suspected enlarged AG by imaging tests [3]. Other reports manifest that some EUS features can be associated with malignancy (e.g., abnormal shape of the AG), but the data are limited [12,13].

The primary aim of this multicenter study was to analyze the diagnostic yield and safety of EUS-TA in the study of AG. Our secondary aims were to determine predictors of malignancy, and to create a preclusive predictive model of malignancy.

## Methods

### Patients and study characteristics

This was a retrospective study, consisting of a noncomparative review of a nationwide database involving all Spanish hospitals experienced in EUS-TA of AG. All partners of the Spanish Group of Endoscopic Ultrasound were invited by mail to participate. Finally, 17 Spanish centers accepted the invitacion. Inclusion period: from April 2003 to April 2016. Inclusion Criteria: All AGs (right and left) punctured by EUS during the inclusion period. Exclusion criteria: loss of contact or information after the procedure. A CRF was designed by the IP center and recollected as a database from the other centers.

The following variables were reviewed: demographic details, clinical data, staging procedures, endoscopic findings, technical details, cytological data, medication with potential risk-bleeding, follow-up data, incidents, and AEs. All imaging parameters were reviewed and taken from the original written reports. This project was developed in accordance with the ethical approval of the Institutional Review Board (Comité Ético de Investigación Clínica, Hospital Universitari de Bellvitge, code PR344/15, 7 january 2016).

### EUS guided TA technique

**Fig 1:**
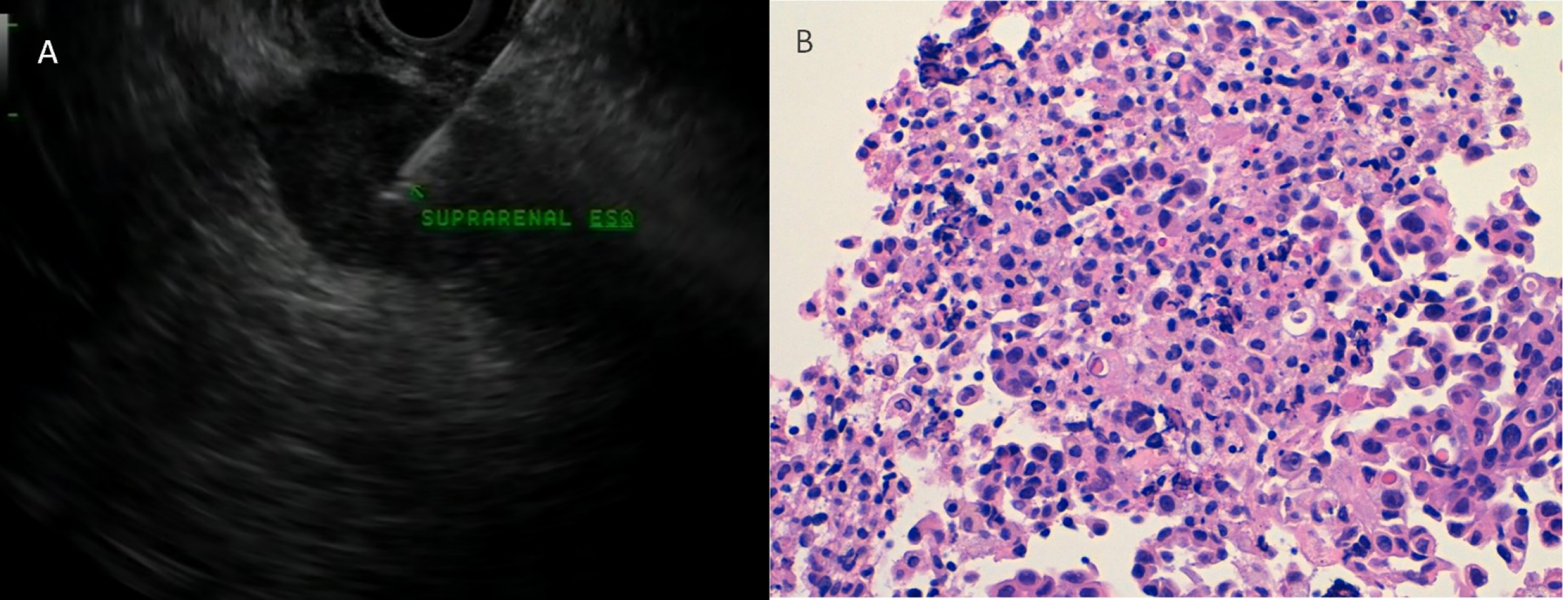
A 63-year-old woman with adenocarcinoma of the lung and a suspicous enlarged adrenal gland. **A** Endoscopic ultrasound-guided fine-needle aspiration of the adrenal gland with a dominant nodule. **B** Adenocarcinoma of the adrenal gland histopathology. H&E, orig. mag. ×400. Courtesy of Dr. Isabel Catala.

All patients provided written informed consent previous to the procedure. All EUS-guided TA were performed by an experienced endosonographer. Deep sedation was assisted by endoscopist or anesthesiologist, depending on the sedation protocol of each center. A linear array echoendoscope (Olympus GF-UCT140-AL5 or UCT-180, Pentax EG-3870UTK or EG-3270UK, Fujifilm EG-580UT) was used to identify and puncture the suspected AG. Left AGs were evaluated from the stomach, and right AGs from the duodenum. EUS-guided puncture was performed using a fine needle (25 or 22G in size), either cytological (EchoTipUltra, Cook; ExpectEndoscopic Ultrasound Aspiration Needle, BostonSC; BeaconFNA exchange system, Medtronic-Covidien) or cytohistopathological (EchoTip ProCore, Cook; SharkCoreFNB exchange system, Medtronic-Covidien). Color Doppler imaging was used to avoid interposal vessels. The suction technique applied, if any (stylet slow-pull vs. standard suction), and type and size of needle were items selected at the discretion of the endosonographer. In patients with antiplatelet or anticoagulant therapy, the recommendations of the international guidelines were followed (i.e., European Society of Gastrointestinal Endoscopy or British Society of Gastroenterology, 2008) [14]. EUS-guided FNA of a left AG example is shown in Fig 1.

### EUS image characteristics

**Fig 2:**
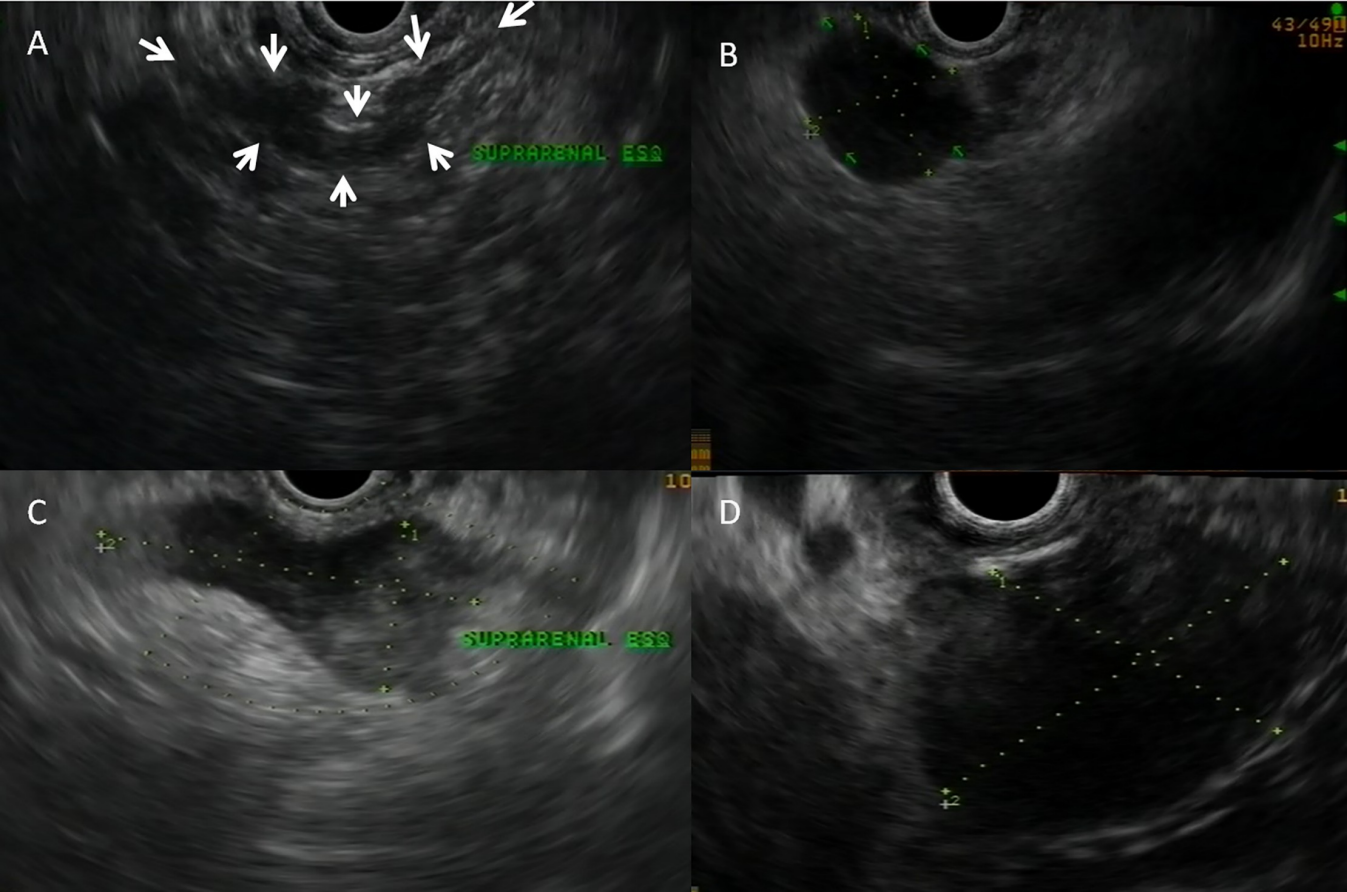
EUS images of four adrenal gland morphology stablished patterns. **A** Usual ‘seagull shape’. **B** Nodule located in a lateral wing. **C** Global enlargement conserving the ‘seagull shape’. **D** Variegated morphology.

The AGs were classified into four types based on morphology and shape: (i) seagull shape; (ii) seagull shape with a nodule or enlargement of one limbs, (iii) global enlarged AG preserving the seagull shape, (iv) variegated morphology (Fig 2).

The following measurements were made according to the EUS image: AG length was the greatest extension between the end of the two wings, and the thickness was measured at the widest body of the AG. The echogenicity was classified as hypoechoic, hyperechoic, or isoechoic. The echo-pattern was divided into homogeneous and heterogeneous.

Finally, the suspected diagnosis was collected according to the experienced endosonographer’s judgement: suspicion of benignity, malignancy, or undetermined AG.

### Cytological examination

Samples were prepared in the procedure room as a rapid on-site evaluation (ROSE) or without ROSE, depending of each center’s protocol. The cytology was categorized according to the following groups: malignant, suspicion of malignancy, benign, atypical cells, and insufficient sample. All specimens were examined by a cytopathologist for a definitive diagnosis. Immunohistochemistry data were recorded. Final diagnoses were: normal AG, adenoma of AG, pheochromocytoma, and metastasis of known tumour or unknown primary tumor.

### Imaging procedures

Computed tomography (CT), scan evaluation: the morphology of the AG was assessed as normal or pathological, according to the radiologists of each center (increase of global size, presence of nodules or masses, irregular margins). The largest AG diameter (mm) was recorded.

Positron emission tomography (PET) scan study: the SUV (standard uptake value) of the affected AG was analyzed in comparison with the baseline collection from the patient's liver in the same study according to one of the following options: hypercaptation, normocaptation, or hypocaptation.

### Statistical analysis

Nominal categorical variables were described by the number of cases, the percentage of the total for category, and the number of missing data. Ordinal categorical variables were described as nominal categorical, or by the number of cases, median, interquartile range, and number of missing data. Continuous variables were described by the number of cases, mean, standard deviation, median, first and third quartile, and number of missing data.

Diagnostic accuracy of echoendoscopist’s suspicion was analysed using cytology obtained by EUS-TA, as gold standard. Sensivity (S), specifity (Sp), predicte values (PV) and area under the ROC curve, were estimated.Diagnostic performance of EUS-guided TA was defined as the total amount of conclusive citopathology results (malignancy plus benignity) excluding the inconclusive results (atypical cells, suspect, insufficient sample).

Dependent variable of the study was cytology result. Independent variables of the study were CT scan morphology, PET scan uptake, echogenicity, echo pattern, endoscopist suspicion, AG size, AG shape and puncture technique features.

A multivariate logistic model was estimated to cytology result. Odds ratios (OR) and confidence interval at 95% were reported. The model was fitted using stepways selection focusing on lowering the Akaike Information Criteria because it penalizes the model for complexity. Model goodness of fit was evaluated using the Chi-square statistic to compare observed vs expected. To evaluate discrimination, the c statistic of Hosmer-Lemeshow was used, which for dichotomous outcome variables is equivalent to the area under the ROC curve (Receiver Operating Characteristic). The Hosmer-Lemeshow test is a goodness of fit especially for risk prediction models. A goodness of fit test measures how well your data fits the model. This test is usually accompained by a classifcation table of observed and expected frequencies.

Whenever possible, estimators were accompanied by a confidence interval at 95%. Statistical significance was set at a probability level <0.05. The statistical package used to process the data and carry out the analysis was R version 3.2.5 for Windows. (S1 and S2 Dataset).

## Results

### Patient data

A total of 200 patients and 204 AGs were collected, with a mean age of 65 years (SD 9.7), mostly men (152; ratio 3.1). The most common indication of EUS-guided puncture was a patient with primary pulmonary tumor, and the left AG was more usual. Demographic and clinical characteristics are detailed in Table 1.

**Table 1:** Clinical and demographic characteristics.

Clinical and demographics	
**n = 200 P / 204 AG**	
Age: mean (SD)	65.47 (9.7)
**Gender/Sex, n (%)**	
Men	152 (76)
Women	48 (24)
**Primary tumor, n (%)**	
Lung	140 (70.0)
Another^a^	38 (19.0)
Unknown	22 (11.0)
**AG, n (%)**	
Left	190 (93.14)
Right	14 (6.86)
**Antithrombotics, n** APA DOAC	
	38 (19)
	21 (10.5)

APA, antiplatelet agents; P, Patients; AG, Adrenal gland

DOAC, direct oral anticoagulant; SD, Standard deviation

Another tumors: biliopancreatic (8); liver (4); digestive tract (8); adrenal gland (1); gynecological (7); hematological (4); kidney (3); melanoma (1); granulomatous disease (1); sarcoma (1).

### Endoscopic and imaging procedures

Technical aspects of EUS-guided puncture are summarized in Table 2. The standard suction, with syringe, was the most frequently used modality (66.18%), and the median number of passes was 2.0 (interquartile range: 1.0 to 3.0). Most of the deep sedations were controlled by an anesthesiologist (67.16%), and the rest by endoscopists (32.35%).

**Table 2:** Puncture technique details.

**Needle type, n (%)**	
Cytological needle	153 (75.0)
Cytohistological needle	31 (15.20)
Missing	20 (9.80)
**Needle size, n (%)**	
19-G	7 (3.43)
22-G	132 (64.71)
25-G	57 (27.94)
Other	3 (1.47)
Missing	5 (2.45)
**Aspiration technique, n (%)**	
Suction-syringe	135 (66.18)
Without syringe (slow-pull)	53 (25.98)
Others	6 (2.94)
Missing	10 (4.94)
**Number of passes: median (IQR)**	2.0 (1.0–3.0)

G, Gauges; IQR: inter quartile range

Data related to the imaging for the staging are detailed in Table 3. A pathological morphology in the CT scan report and a hypercaptation in the PET scan were the most common findings. Information related to the EUS findings is reflected in Table 4: hypoechogenicity, heterogeneous pattern, and a seagull shape with enlargement of one of the limbs, or variegated were noteworthy.

**Table 3:** Staging of imaging procedures.

**CT scan morphology, n (%)**	
Normal	38 (18.63)
Pathological	145 (71.08)
Missing	21 (10.29)
**PET scan uptake, n (%)**	
Hypercaptation	96 (47.06)
Normocaptation	16 (7.84)
Hypocaptation	4 (1.96)
Missing	88 (43.14)

CT, computed tomography; PET, positron emission tomography

**Table 4:** Endoscopic ultrasound findings.

**Echogenicity, n (%)**	
Hyperechoic	9 (4.41)
Hypoechoic	179 (87.75)
Isoechoic	9 (4.41)
Missing	7 (3.43)
**Echo pattern n (%)**	
Homogeneous	79 (38.73)
Heterogeneous	113 (55.39)
Missing	12 (5.88)
**AG shapes, n (%)**	
Usual seagull shape	3 (1.47)
Seagull shape with enlargement of one of its limbs	84 (41.18)
Seagull shape with a global enlargement	46 (22.55)
Variegated morphology	68 (33.33)
Other	3 (1.47)
**Endoscopist Suspicion, n (%)**	
Benignity	25 (12.25)
Malignancy	141 (69.12)
Undetermined	37 (18.14)
Missing	1 (0.49)

AG, Adrenal gland; EUS, Endoscopic ultrasound

The diagnostic yield was 91.17%: malignant plus benign final diagnostic results. Samples were nondiagnostic in 8.83% including atypia (0.98%), suspicious but not conclusive (2.94%), and insufficient (4.90%) (Table 5). No significant differences between FNA or FNB needles were found.

**Table 5:** Results of EUS-guided puncture and adverse events.

**Citopathology n (%)**	
Malignancy	122 (59.80)
Benignity	64 (31.37)
Atypical cells	2 (0.98)
Suspect	6 (2.94)
Insufficient sample	10 (4.90)
**Diagnostic performance n (%)**	186 (91.17)
**Adverse effects n (%)**	1 (0.49)

**Fig 3:**
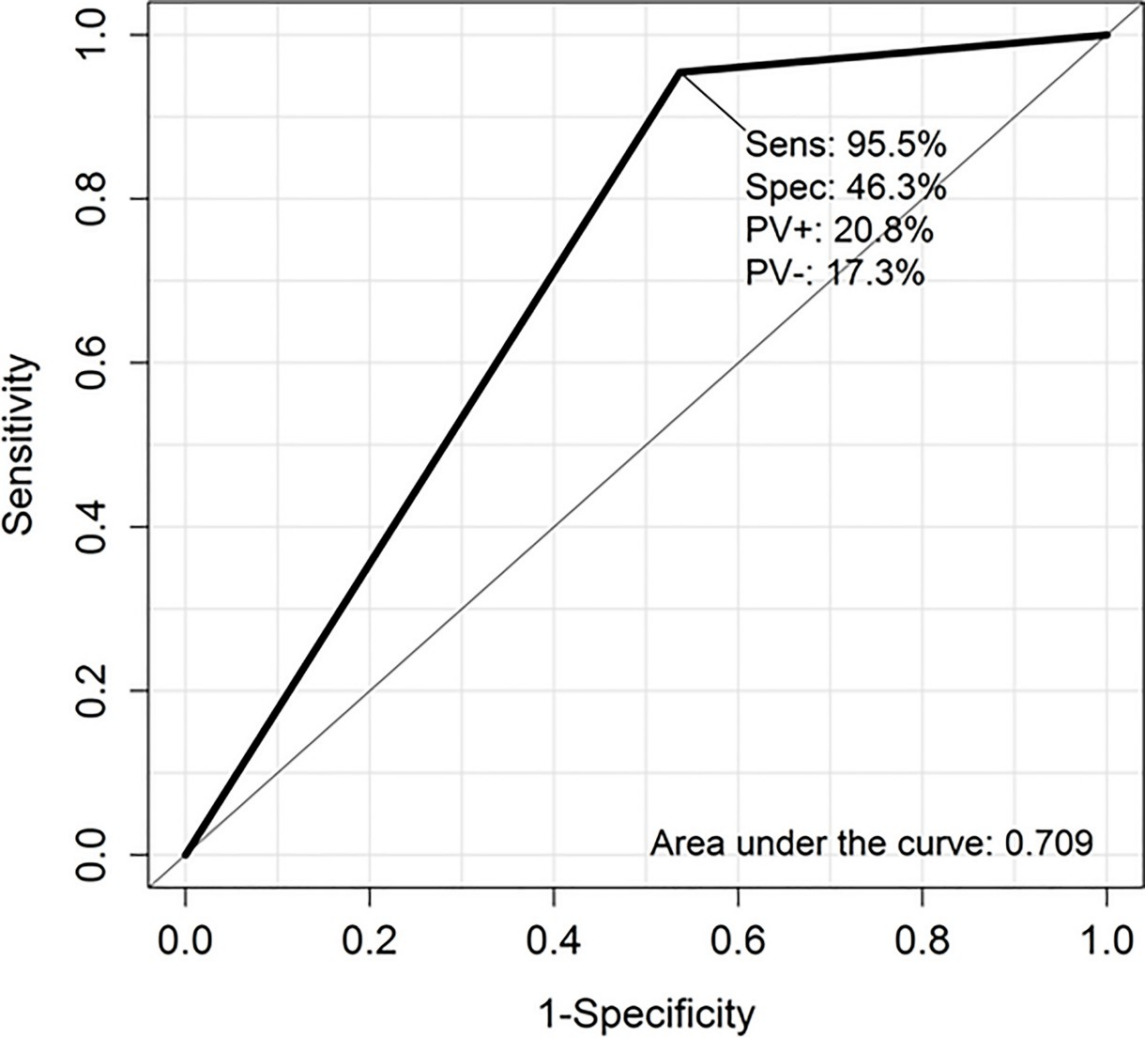
ROC curve showing specificity (Spec) and sensitivity (Sens) of the diagnostic accuracy. Area under the curve: 0.70. Predictive value positive (PV+), predictive value negative (PV-).

Endosonographer suspicion based on the EUS image, was classified as benign or malignant in 81% of EUS procedures (19% was undetermided). Diagnostic accuracy of this was 68%. In Fig 3 are detailed S, Sp and PVs for the endosonographer suspicion using cytology, obtained by EUS-TA, as gold standard.

### Safety

Only one case of self-limiting post-procedural fever was detected, with negative blood tests. It is of note that 10.5% AGs were treated with anticoagulant therapy and 19% were treated with antiplatelet therapy, without any cases of bleeding. Two cases of unknown pheochromocytoma were punctures without concomitant hypertensive crises during the EUS procedure. In the majority of cases, no specific study for pheochromocytoma was performed previous to the endoscopy procedure. No procedure-related mortality was reported.

### Univariate and multivariate analysis

A lower age, pathological image in CT scan, hypercaptation in PET scan, variegated AG shape, size > 30mm, heterogeneous EUS pattern, and suspicion of malignancy by the endoscopist were statistically associated with a malignant cytology result. In contrast, hypoechoic AG and lateral nodule AG shape were not associated with malignancy (Table 6).

**Table 6:** Variables associated with malignancy. Univariate analysis (n-186).

	Malignants (122)	Benigns (64)	OR, CI 95%	p-value
**Age, mean (SD)**				
Age	64.0 (10.01)	67.6 (9.07)	0.96 (0.93–0.99)	0.018
**Sex, n(%)**				
Men	27 (22.1%)	23 (35.9%)	Reference	
Women	95 (77.9%)	41 (64.1%)	1.97 (1.00–3.85)	0.049
**CT scan morphology, n**^**a**^ **(%)**				
Normal	17 (15.3)	20 (35.1)	Reference 2.96 (1.40–6.38)	
Pathological	94 (84.7)	37 (64.9)		0.005
**PET scan uptake, n**^**b**^ **(%)**				
Normcaptation	3 (4.29)	12 (35.3)	Reference 11.4 (3.23–56.5)	
Hypercaptation	67 (95.7)	22 (64.7)		<0.001
**AG Morphologies, n**^**c**^ **(%)**				
Usual seagull shape	0 (0.0)	2 (3.12)	NA Reference 1.21 (0.57–2.65) 2.99 (1.37–6.91)	
Enlargement of one of its limbs	46 (37.7)	32 (54.2)		
Increase of global size	28 (23.0)	16 (27.1)		0.622
Variegated morphology	48 (39.3)	11 (18.6)		0.005
**AG size > 30mm, n**^**d**^ **(%)**				
No	51 (44.3)	38 (64.4)	Reference 2.27 (1.19–4.38)	
Yes	64 (55.7)	21 (35.6)		0.013
**Echo pattern, n**^**e**^ **(%)**				
Homogeneous	42 (35.9)	34 (54.8)	Reference 2.16 (1.15–4.07)	
Heterogeneous	75 (64.1)	28 (45.2)		0.016
**EUS Suspicion, n**^**f**^ **(%)**				
Benign	5 (4.55)	19 (46.3)	Reference 17.3 (6.17–58.0)	
Malignant	105 (95.5)	22 (53.7)		<0.001
**EUS Indication, n**^**g**^ **(%)**				
Lung neoplasia	90 (75.6%)	40 (63.5%)	Reference	
Other neoplasia	22 (18.5%)	13 (20.6%)	0.75 (0.35–1.68)	0.479
Unknown neoplasia	7 (5.88%)	10 (15.9%)	0.32 (0.11–0.89)	0.030

OR, *Odds Ratio*; CI, confidence interval; SD, standard deviation; CT, computed tomography; PET, Positron emission tomography; AG, Adrenal gland; EUS, Endoscopic ultrasound; NA, not applicable

Numer of missing values

a 18 (9.7%)

b 82 (44%)

c 3 (1.6%)

d 12 (6.5%)

e 7 (3.8%)

f 35 (18.8%) and

g 4 (2.2%).

A predictive model for malignancy was formulated using multivariate logistic regression (Table 7 and S1 Fig). The final model included two demographic factors, age and sex, and two factors measured by EUS, echo pattern and AG shape. Among the factors ruled out were echogenicity and AG size > 30mm. Neither CT scan morphology nor PET scan uptake were considered for inclusion in the model. The selection of variables was taken in a count depending the objectivity and clinical impact for each one. Although the variable 'EUS suspicion' was the most predictive parameter in the univariate analysis, it had not been 'included' in the multivariate analysis because it is a subjective variable and operator-depending.

**Table 7:** Multivariate logistic model of malignancy (n-174).

Variables	OR IC 95%	p-value
Constant	2.39 (0.72–8.02)	0.15584
Female	0.44 (0.20–0.94)	0.03343
Age (5 years periods)	0.77 (0.63–0.93)	0.00986
Heterogeneous	2.56 (1.27–5.27)	0.00917
Global enlarged AG	1.67 (0.72–4.01)	0.24393
Variegated AG shape	3.25 (1.41–7.99)	0.00746

AG, adrenal gland.

**Fig 4:**
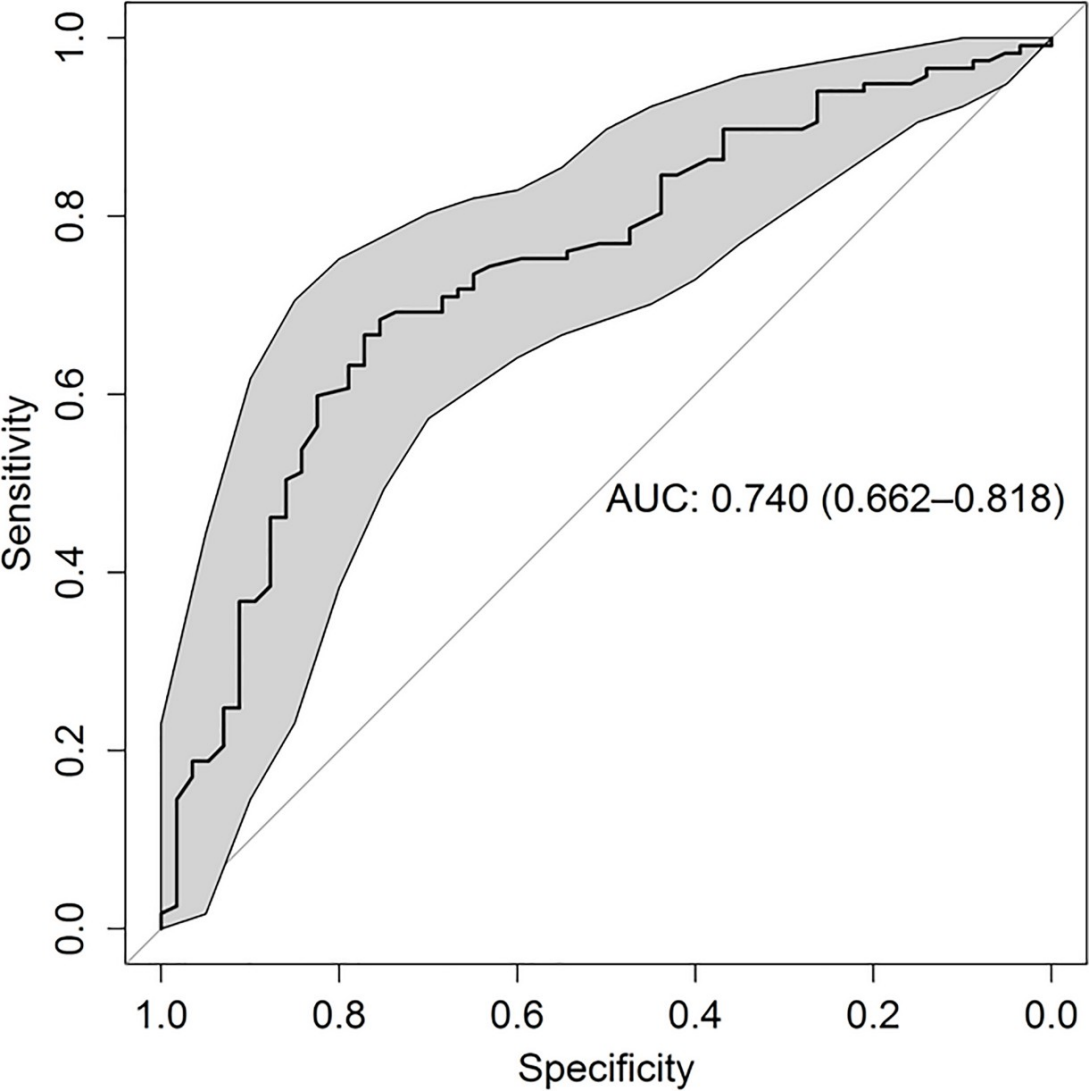
ROC curve of malignancy model. Estimator of the area under the curve: 0.74 CI 95% [0.66–0.82]. Area under the ROC curve was estimated using the final model with the following factors: age, sex, echo pattern and adrenal gland shape.

The derived prediction model had good discrimination with area under the ROC curve 74% 95%CI 66% to 82% (Fig 4). Calibration was also acceptable, with good observed/expected ratio for all strata of predicted risk. In Table 8, patient’s malignacy probability (calculated with the estimated model) were grouped by decils and the observed event rates in each decil are presented. In the first decil of risk, with a probability of malignacy that ranged from 0.17 to 0.43, 6 of 18 malignacy cases were observed; in the last decil of risk, with a probability of malignacy that ranged from 0.88 to 0.97, 17 of 18 malignacy cases were observed. Hosmer and Lemeshow goodness of fit test suggested a rasonable fit of data to the estimated model (Chi2 5.8 p-value = 0.6689). Finally, a cut-off of malignacy probability defined two groups of patients: those with a postive result (probability of malignacy equal or above 0.4) and those with a negative result (probability of malignacy below 0.4). Among positive patients 112 of 161 cases malignacy was found and among negative patients 5 of 13 cases malignacy.

**Table 8:** Observed and model predicted decils of malignancy.

**Probability of malignancy**	**No malignancy**	**Malignancy**
[0.166,0.432]	12 (67%)	6 (33%)
(0.432,0.5]	9 (47%)	10 (53%)
(0.5,0.558]	6 (40%)	9 (60%)
(0.558,0.619]	10 (56%)	8 (44%)
(0.619,0.705]	7 (41%)	10 (59%)
(0.705,0.755]	4 (24%)	13 (76%)
(0.755,0.792]	2 (11%)	16 (89%)
(0.792,0.848]	2 (12%)	15 (88%)
(0.848,0.876]	4 (24%)	13 (76%)
(0.876,0.971]	1 (6%)	17 (94%)
AG*	**No pathological**	**Pathological**
Positive (p≥0.4)	49	112
Negative (p<0.4)	8	5

* If the probability of malignancy is >0.40, we would consider the adrenal gland (AG) as pathological.

## Discussion

This was a nationwide multicenter study with the largest number of cases of AG punctured by EUS-TA, FNA or FNB, to date. All procedures were performed by a dedicated endosonographer expert in the diagnosis and staging of digestive and lung cancer. This study supports the concept, previously introduced by other authors, that EUS-TA is a safe and accurate, minimally invasive technique, and it may be considered as the first choice in the study of suspicion in AGs, basically when this information can really help to the treatment strategy [3,11–13].

Nowadays, most imaging tests (PET, CT) are very powerful and reliable methods for the staging of many tumors. But, specifically in the study of AGs, these tests still have some limitations, and there remains the need to obtain biological material for pathological study. In the radiological imaging literature, there is an evident limitation in the accuracy of these imaging tests, as there is a considerable number of false-negatives (metastases misdiagnosed as adenomas) and false-positives (adenomas classified as metastases) [2,15,16]. In the same line, this current study has revealed a considerable percentage of cases with a benign profile in the EUS image that obtained a result of malignancy in cytology. This suggests that it is still essential to obtain a pathological sample to confirm a suspicion of metastasis that will produce a change in the therapeutic attitude: radical surgical treatment is ruled out and treatment with systemic chemotherapy or a palliative option are chosen instead.

There are many reasons to consider EUS-TA an attractive technique for the evaluation of suspicious AGs. One obvious advantage of this endosonographer technique is in a lung cancer scenario, where the EUS-TA can combine evaluation of a mediastinal tumor and of an enlarged AG during the same procedure, plus without any irradiation [3].

A recent review on the role of EUS-FNA in the diagnosis of adrenal lesions considered 17 original articles (seven case reports, ten case series) including a total of 416 cases [11]. The average AG size was 25.9mm; FNA needle size used was 19, 22, and 25 G, and the average number of passes was two. A total of 25 right AGs were documented, no major AEs were reported, and only one adrenal hemorrahge case was detected [17]. Regarding EUS image findings, it is of note that a hypoechoic feature (usually related to malignancy) was not associated with a malignant cytological result [11,18]. In the same line, Eloubeidi et al reported interesting findings: on a multivariable analysis an altered AG shape was a significant predictor of malignancy, whereas a size >30mm and hypoechoic nature were not [13]. Based on size >30mm alone, EUS had limited accuracy (68%). This finding is in striking contrast to the strong correlation of this variable with the accuracy of the imaging tests [15].

This nationwide study reinforces the hypothesis that EUS-TA of AGs is a reproducible and feasible technique, clearly assumable in endoscopy units that routinely perform this procedure. The high diagnostic yield, with a small percentage of nondiagnostic biopsies and insufficient samples, is similar to the data that come from an expert single-center in this area [10,13]. No differences were encountered between cytological or cytohistological needles, and the rate of confirmed malignancy was high, explained by the elevated proportion of lung cancer staging included in this study. All patients were managed according to the cytological results obtained by EUS-TA, a well-accepted method by oncologist and surgeons. Another strength offered by this multicenter study is that it provides the largest case series of right AG punctures via transduodenal approach with a similar prevalence (6%), compared with the recent review by Patil et al [11,19].

It is important to note that some variables related to the EUS image (variegated AG shape, heterogeneous echo-pattern, size >30mm) were clearly associated with the malignancy diagnosis, but the most accurate variable of EUS image was the endosonographer’s opinion. When it was analyzed using the cytology, obtained by EUS-TA as a gold standard, the endoscopist’s opinion showed an excellent S, good diagnostic accuracy but low Sp. This highlights the observation that EUS is an operator-depenent procedure, and the intuition of the endoscopist is more credible and valuable than each variable taken separately.

As in previous papers, a hypoechoic EUS feature was not associated with a diagnosis of malignancy by EUS-FNA [13,18]. In our opinion, the repetition of this finding in this study can be considered a validation of the hypothesis that a hypoecohoic AG does not mean a malignant AG, and does not exclude the performance of an EUS-TA.

The presented model allows estimation of the probability of a diagnosis of malignancy. The best variables for the predictive multivariate logistic model were age, sex, echo pattern, and AG shape determined by EUS. This model has an excellent S, good positive PV and negative PV, but low Sp. Therefore, the probability of predicting the positive pathological AG is very high, but the probability of predicting how negative the non-pathological AG is, is low. It should be borne in mind that, due to a diagnosis confirmation bias, estimation of S and Sp is very likely to be high and low, respectively.

In addition, both the probability of having a pathological AG and presenting a positive result as well as the probability of having a non-pathological AG and presenting a negative result are > 75%. But these results are determined by the prevalence; if the estimated prevalence (63% of malignant AG confirmed by EUS-TA in this study) does not reflect the usual prevalence of pathological AG, the PPV value and the NPV value will not be credible and should be ruled out.

No serious AEs were documented in this current study and other studies of EUS-FNA in the AG, in contrast with the AE rate related to percutaneous biopsy [7–9]. No other organ except the gastric or duodenal wall is punctured to access the AG. In this study no 19G needles were used; only ‘fine’ (22 or 25 G) needles were utilized. This may have minimized AEs associated with puncture, such as bleeding and perforation. The use of cytohistological FNB needles (‘core’, with a more agressive tip design) was not related to more AEs.

Regarding the risk of puncture of a previously undicovered pheochromocytoma, in this study this occurred in two cases that were diagnosed with EUS-TA in which no previous urine studies were performed. Although some authors recommend that urine studies should be performed prior to attempting an AG guided-puncture, EUS-FNA of pheochromocytomas and paragangliomas has been reported as safe [20–21]. Perhaps it would be safer to rule out pheochromocytoma prior to puncturing an AG in patients without cancer, but in cases with known primary malignancy, this may not be necessary [22].

### Limitations

Our study has some limitations. Firstly, there is variability among the different centers, with varying levels of expertise among the endosonographers; this may have instroduced some bias in the results (e.g., technical aspects, measuring AG, interpretation of AG shape). Secondly, the high proportion of lung cancers cases may have introduced a selection bias in the study population. Thirdly, a retrospective study is associated with a lack of patient contact and missed follow-ups; this is associated with a loss of some AEs.

There is also the limitation of the predictive model: the number of data used, although they are all available to date, did not allow us to reserve part of these to perform a validation of the model. Validation in a different sample of the development of the model presented here is pending. Although the statistics to evaluate the predictive capacity of the model are quite satisfactory, they may be under- or over-estimated due to a diagnostic confirmation bias or because of a higher or lower than expected prevalence of malignancy in the sample.

In conclusion, EUS-TA of the AG is a safe, minimally invasive procedure, with an excellent diagnostic yield. Some EUS image features are associated with a diagnosis of malignancy, and this study suggests the possibility of developing a malignancy-predictive model for us prior to the EUS procedure.

## Supporting information

S1 Fig Multivariate logistic model of malignancy. Variables and their statistical association with the malignancy risk. Adrenal gland (AG).
(TIF)

S1 Dataset Minimal data set. (DOCX)

S2 Dataset Excel EUS TA adrenal data set. (XLSX)

## References

[1] Chapman GS, Kumar D, Redmond J 3rd, Munderloh SH, Gandara DR. Upper abdominal computerized tomography scanning in staging non-small cell lung carcinoma. Cancer 1984;54:1541–1543. 6478395 10.1002/1097-0142(19841015)54:8<1541::aid-cncr2820540812>3.0.co;2-n

[2] Burt M, Heelan RT, Coit D, McCormack PM, Bains MS, Martini N et al Prospective evaluation of unilateral adrenal masses in patients with operable non-small- cell lung cancer. Impact of magnetic resonance imaging. J Thorac Cardiovasc Surg 1994;107:584–588. 8302078

[3] Schuurbiers OC, Tournoy KG, Schoppers HJ, Dijkman BG, Timmers HJ, de Geus-Oei LF et al EUS-FNA for the detection of left adrenal metastasis in patients with lung cancer. Lung Cancer 2011;73:310–315. 10.1016/j.lungcan.2010.12.019 21277038

[4] Brady MJ, Thomas J, Wong TZ, Franklin KM, Ho LM, Paulson EK. Adrenal nodules at FDG PET/CT in patients known to have or suspected of having lung cancer: a proposal for an efficient diagnostic algorithm. Radiology 2009;250:523–530. 10.1148/radiol.2502080219 19188319

[5] Gupta NC, Graeber GM, Tamim WJ, Rogers JS, Irisari L, Bishop HA. Clinical utility of PET-FDG imaging in differentiation of benign from malignant adrenal masses in lung cancer. Clin Lung Cancer 2001;3:59–64. 14656394 10.3816/clc.2001.n.019

[6] Okada M, Shimono T, Komeya Y, Ando R, Kagawa Y, Katsube T et al Adrenal masses: the value of additional fluorodeoxyglucose-positron emission tomography/computed tomography (FDG-PET/CT) in differentiating between benign and malignant lesions. Ann Nucl Med 2009;23:349–354. 10.1007/s12149-009-0246-4 19340526

[7] Mody MK, Kazerooni EA, Korobkin M. Percutaneous CT-guided biopsy of adrenal masses: immediate and delayed complications. J Comput Assisted Tomogr 1995;19:434–439. 10.1097/00004728-199505000-00017 7790554

[8] Gillams A, Roberts CM, Shaw P, Spiro SG, Goldstraw P. The value of CT scanning and percutaneous fine needle aspiration of adrenal masses in biopsy-proven lung cancer. Clinical Radiology 1992;46:18–22. 1643776 10.1016/s0009-9260(05)80027-3

[9] Harisinghani MG, Maher MM, Hahn PF, Gervais DA, Jhaveri K, Varghese J et al Predictive value of benign percutaneous adrenal biopsies in oncology patients. Clin Radiol 2002;57:898–901. 12413913 10.1053/crad.2002.1054

[10] Eloubeidi MA, Black KR, Tamhane A, Eltoum IA, Bryant A, Cerfolio RJ. A large single-center experience of EUS-guided FNA of the left and right adrenal glands: diagnostic utility and impact on patient management. Gastrointest Endosc. 2010;71:745–753. 10.1016/j.gie.2009.10.022 20156622

[11] Patil R, Ona MA, Papafragkakis C, Duddempudi S, Anand S, Jamil LH. Endoscopic ultrasound-guided fine-needle aspiration in the diagnosis of adrenal lesions. Ann Gastroenterol. 2016; 29:307–311. 10.20524/aog.2016.0047 27366030 PMC4923815

[12] DeWitt J, Alsatie M, LeBlanc J, McHenry L, Sherman S. Endoscopic ultrasound-guided fine-needle aspiration of left adrenal gland masses. Endoscopy 2007;39:65–71. 10.1055/s-2006-945042 17252463

[13] Eloubeidi MA, Seewald S, Tamhane A, Brand B, Chen VK, Yasuda I et al EUS-guided FNA of the left adrenal gland in patients with thoracic or GI malignancies. Gastrointest Endosc 2004;59:627–633. 15114304 10.1016/s0016-5107(04)00296-2

[14] Veitch AM, Baglin TP, Gershlick AH, Harnden SM, Tighe R, Cairns S et al Guidelines for the management of anticoagulant and antiplatelet therapy in patients undergoing endoscopic procedures. Gut. 2008;57(9):1322–9. 10.1136/gut.2007.142497 18469092

[15] Porte HL, Ernst OJ, Delebecq T, Métois D, Lemaitre LG, Wurtz AJ. Is computed tomography guided biopsy still necessary for the diagnosis of adrenal masses in patients with resectable non-small-cell lung cancer? Eur J Cardiothorac Surg 1999;15:597–601. 10.1016/s1010-7940(99)00047-0 10386403

[16] Xu B, Gao J, Cui L, Wang H, Guan Z, Yao S et al Characterization of adrenal metastatic cancer using FDG PET/CT. Neoplasma. 2012;59:92–9. 22103902 10.4149/neo_2012_012

[17] Haseganu LE, Diehl DL. Left adrenal gland hemorrhage as a complication of EUS-FNA. Gastrointest Endosc 2009;69:e51–2. 10.1016/j.gie.2008.09.035 19152908

[18] Martinez M, LeBlanc J, Al-Haddad M, Sherman S, DeWitt J. Role of endoscopic ultrasound fine-needle aspiration evaluating adrenal gland enlargemenr or mass. World J Nephrol 2014;3:92–100 10.5527/wjn.v3.i3.92 25332900 PMC4202496

[19] Serra K, Llatjos R, Catalá I, Gornals JB. Adrenal metastasis of hepatocellular carcinoma diagnosed by endoscopic ultrasound-guided fine-needle aspiration of the right adrenal gland. Rev Esp Enferm Dig. 2017;109:378 28480731

[20] Stelow EB, Debol SM, Stanley MW, Mallery S, Lai R, Bardales RH. Sampling of the adrenal glands by endoscopic ultrasound-guided fine-needle aspiration. Diagn Cytopathol 2005;33:26–30 10.1002/dc.20273 15945088

[21] Akdamar MK, Eltoum I, Eloubeidi MA. Retroperitoneal paraganglioma: EUS appearance and risck associated with EUS-guided FNA. Gastrointest Endosc 2004; 60:1018–1021 15605027 10.1016/s0016-5107(04)02218-7

[22] Grumbach MM, Biller BM, Braunstein GD, Campbell KK, Carney JA, Godley PA et al Management of the clinically inapparent adrenal mass (“incidentaloma”). Ann Intern Med 2003;138:424–429 12614096 10.7326/0003-4819-138-5-200303040-00013

