# Supplementary figures and images for: EUS-guided tissue acquisition in the study of the adrenal glands: Results of a nationwide multicenter study
Source: PLoS One. 2019 Jun 6;14(6):e0216658. doi: 10.1371/journal.pone.0216658 (PMC6553722; doi:10.1371/journal.pone.0216658)

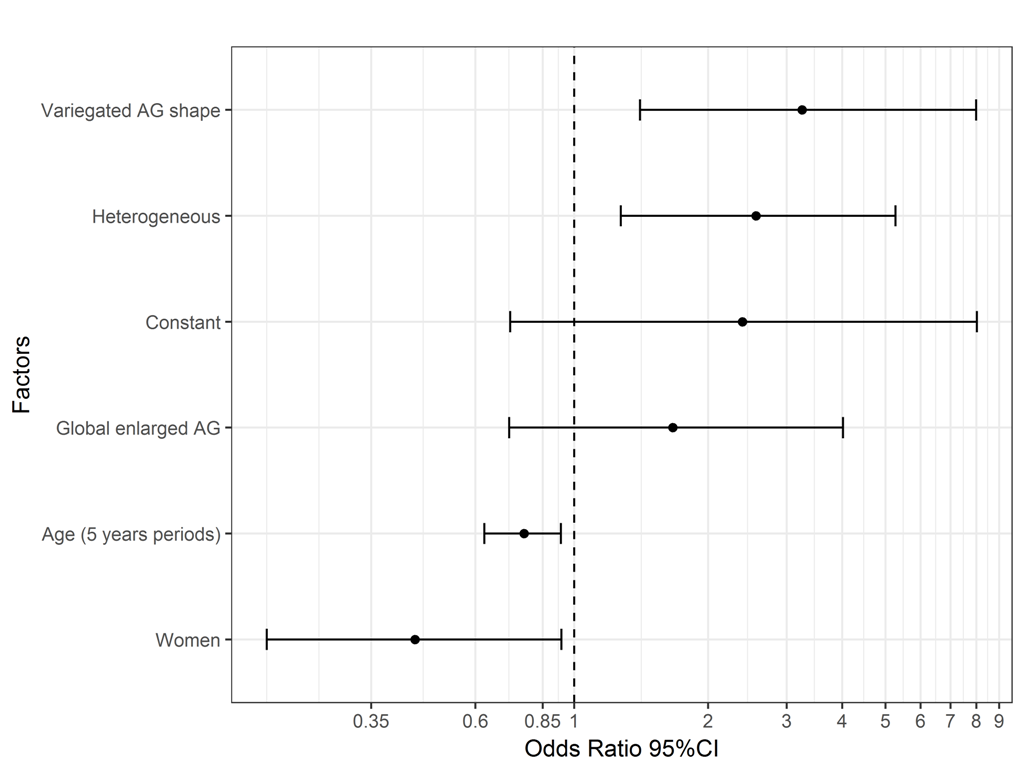

Supplement: S1 Fig — Variables and their statistical association with the malignancy risk. Adrenal gland (AG). (TIF) [file pone.0216658.s001.tif]
