# Supplementary material for: EUS-guided tissue acquisition in the study of the adrenal glands: Results of a nationwide multicenter study
Source: PLoS One. 2019 Jun 6;14(6):e0216658. doi: 10.1371/journal.pone.0216658 (PMC6553722; doi:10.1371/journal.pone.0216658)
Supplement: S1 Dataset — (DOCX) [file pone.0216658.s002.docx]

**Statistical analysis of ‘EUS-Guided tissue acquisition in the study of the adrenal glands: Results of a nationwide multicenter study**.

IP Joan Gornals

Hospital Universitari de Bellvitge

Statistic: Cristian Tebé

2.0 - october 2016

**Content**

[Analysis 4](#_Toc465259895)

[Tables and figures 5](#_Toc465259897)

[Objective 1: cytology 9](#_Toc465259898)

[Objective 2: Evolution 10](#_Toc465259899)

[Objective 3: clinical impact 13](#_Toc465259900)

[Objective 4: benign profile 15](#_Toc465259901)

[Objective 5: Predictive model of malignancy 16](#_Toc465259902)

[Annex: 20](#_Toc465259903)

**Stastistical analysis**

## The variables that collect the demographic and clinical characteristics of the patients will be analyzed based on the type of variable and presented in tables. The nominal categorical variables will be described by means of the number of cases, the percentage with respect to the total by category and the number of absent data. Ordinary categorical variables will be described as nominal categories, or by means of the number of cases, the mean, the interquartile range and the number of absent data. The continuous variables will be described by means of the number of cases, the average, the standard deviation, the median, the first and the third quartile and the number of absent data.

## To compare two categorical variables, the Chi2 test will be used. The comparison of mean between groups will be done through the test t or Wilcoxon test based on the distribution of the continuous variable.

## The variables dependent on the study are the cytology (benign / pathology), clinical evolution (Success / no) and change of clinical attitude (yes / no). The independent variables of the study are the morphology for TAC, PET, USE- ecogenicity, USE- homogeneity, USE-suspect, type, size, technique and number of needle strokes.

## An exploratory form will estimate a multivariate logistics model of malignancy for cytology, mortality and therapeutic change. As predictors, the factors described in the previous section will be used. The results will be presented in the form of a graph with odds ratio (OR) estimates and a confidence interval of 95%.

## Wherever possible, the estimators will be accompanied by a confidence interval of 95%. The statistical significance is set at a probability level <0.05. The statistical package that will be used to handle the data and perform the analysis will be the R version 3.2.5 for Windows.

## Tables and figures

| Table 1.Demographics. | |
| --- | --- |
| **Variables** | **n=200 patients** |
| **Age** | |
| Average (DE) | 65.47 (9.71) |
| Medium (RIQ) | 66.0 (59.0 - 72.0) |
| **Sex** | |
| Man | 152(76.00%) |
| Female | 48 (24.00%) |

DE: standard deviation; RIQ: interquartile range

| Table 2.Clinical (n=200 patients). | |
| --- | --- |
| **Primary Tumor** | |
| Lung | 139 (69.50%) |
| Unknown | 21 (10.50%) |
| Others | 19 (9.50%) |
| Pancreas | 8 (4.00%) |
| Gut | 7 (3.50%) |
| Hepatocarcinoma | 4 (2.00%) |
| Lung + digestive | 1 (0.50%) |
| Missing | 1 (0.50%) |
| **Adrenal** | |
| Right | 11 (5.50%) |
| Left | 189 (94.50%) |
| **Anticoagulant** | |
| Y | 21 (10.50%) |
| N | 179 (89.50%) |
| **Antiaggregant** | |
| Y | 38 (19.00%) |
| N | 161 (80.50%) |
| Missing | 1 (0.50%) |
| **NSAID recent** | |
| Y | 40 (20.00%) |
| N | 158 (79.00%) |
| Missing | 2 (1.00%) |

| Table 3. Imaging (n=204 procedures). | |
| --- | --- |
| **CT** | |
| Normal | 38 (18.63%) |
| Pathol | 145 (71.08%) |
| Missing | 21 (10.29%) |
| **ANR** | |
| Y | 137 (67.16%) |
| N | 66 (32.35%) |
| Missing | 1 (0.49%) |
| **EUS ≥30** | |
| Y | 92 (45.10%) |
| N | 96 (47.06%) |
| Missing | 16 (7.84%) |
| **PET** | |
| Hyper | 96 (47.06%) |
| Normo | 16 (7.84%) |
| Hypo | 4 (1.96%) |
| Missing | 88 (43.14%) |
| **Echo pattern** | |
| Hipoechoic | 179 (87.75%) |
| Hiper | 9 (4.41%) |
| Iso | 9 (4.41%) |
| Missing | 7 (3.43%) |
| **Internal pattern** | |
| Homogenicity | 79 (38.73%) |
| Hetero | 113 (55.39%) |
| Missing | 12 (5.88%) |
| **EUS morphology** | |
| Normal, “seagull” | 3 (1.47%) |
| “Seagull” lateral | 84 (41.18%) |
| “Seagull” global increase | 46 (22.55%) |
| Tumoral | 68 (33.33%) |
| Others | 3 (1.47%) |
| **Endoscopist suspicious** | |
| Benignicity | 25 (12.25%) |
| Malignancy | 141 (69.12%) |
| Undetermined | 37 (18.14%) |
| Missing | 1 (0.49%) |

| Table 4.Techincal variables (n=204 procedures) | |
| --- | --- |
| **Variables** |  |
| **Needle type** | |
| Cytological | 153 (75.00%) |
| Cytohystological | 31 (15.20%) |
| Missing | 20 (9.80%) |
| **Size** | |
| 19-gauge | 7 (3.43%) |
| 20-gauge | 132 (64.71%) |
| 22-gauge | 57 (27.94%) |
| 25-gauge | 3 (1.47%) |
| Missing | 5 (2.45%) |
| **Technique** | |
| FNA using syringe | 135 (66.18%) |
| FNA without syringe | 53 (25.98%) |
| Others | 6 (2.94%) |
| Missing | 10 (4.90%) |
| **Number of passes** | |
| Average (DE) | 2.18 (1.39) |
| Med. (RIQ) | 2.00 (1.00 - 3.00) |
| Missing | 5 (2.45%) |

| Table 5. AP results (n=204 procedures). | |
| --- | --- |
| **Cytology** | |
| Malignancy | 122 (59.80%) |
| Benignicity | 64 (31.37%) |
| Atypical cel | 2 (0.98%) |
| Suspicious | 6 (2.94%) |
| Non diagnostic | 10 (4.90%) |
| **Evolution** | |
| Life | 69 (33.82%) |
| Exitus | 101 (49.51%) |
| Missing | 34 (16.67%) |
| **Clinical impact** | |
| Y | 133 (65.20%) |
| N | 69 (33.82%) |
| Missing | 2 (0.98%) |

The registry has information of 205 adrenals of 200 patients. The information collected from a duplicate patient has been discarded (# 1995018836); leaving the record in 204 adrenals of 200 patients. There are 2 patients (# 577744 and # 645406) ​​with two adrenals deferred over time and 3 patients with the right and left adrenal informed (# 703144, # 613518 and # 398101). The distance between patient punctures # 577744 is 3 years and therefore evaluated as if they were two independent patients.

The average age of the patients is 65 years old where 25% were under 60 years old and another 25 more than 72 years. Of the patients analyzed the male / female ratio was 3 to 1. 69% of the tumors were primary in the lungs and 10% in the primary tumor was unknown (Table 2 describes the rest). 93% of the adrenals were left.

Table 3 describes the characteristics of the extension study. Where most of the suprerenales presented a morphology for pathological TAC (71%), they were hipercaptants (82% of those reported), hypoecoic or hypoecogens (88%) and suspected malignancy for USE (69%).

Table 4 describes the characteristics of the needle and the puncture. Where type of dominant needle was cytological (75%), 22-gauge (64%) and PAAF aspiration with syringe (66%).

Finally, in Table 5, the main outcome measures are described. 92% of the samples allowed to make a diagnosis for cytology and 60% of these were malignant.

## Objective 1: cytology

| Table 6.Factors related to cytology. | | | | | |
| --- | --- | --- | --- | --- | --- |
| **Variables** | Malignant **No. 122** | Benigne **No. 64** | atypical **No. 2** | Suspicious **No. 6** | Non-diag **No. 10** |
| **CT*** |  |  |  |  |  |
| Normal | 17 (13.93%) | 20 (31.25%) | 0 (0.00%) | 0 (0.00%) | 1 (10.00%) |
| Pathol | 94 (77.05%) | 37 (57.81%) | 2 (100.00%) | 5 (83.33%) | 7 (70.00%) |
| Missing | 11 (9.02%) | 7 (10.94%) | 0 (0.00%) | 1 (16.67%) | 2 (20.00%) |
| **ANR**** |  |  |  |  |  |
| Y | 80 (65.57%) | 47 (73.44%) | 1 (50.00%) | 0 (0.00%) | 9 (90.00%) |
| No | 41 (33.61%) | 17 (26.56%) | 1 (50.00%) | 6 (100.00%) | 1 (10.00%) |
| Missing | 1 (0.82%) | 0 (0.00%) | 0 (0.00%) | 0 (0.00%) | 0 (0.00%) |
| **Size ≥30***** |  |  |  |  |  |
| Y | 64 (52.46%) | 21 (32.81%) | 2 (100.00%) | 3 (50.00%) | 2 (20.00%) |
| N | 51 (41.80%) | 38 (59.38%) | 0 (0.00%) | 0 (0.00%) | 7 (70.00%) |
| Missing | 7 (5.74%) | 5 (7.81%) | 0 (0.00%) | 3 (50.00%) | 1 (10.00%) |
| **PET****** |  |  |  |  |  |
| Hyper | 67 (54.92%) | 22 (34.38%) | 0 (0.00%) | 4 (66.67%) | 3 (30.00%) |
| Normo | 3 (2.46%) | 12 (18.75%) | 0 (0.00%) | 0 (0.00%) | 1 (10.00%) |
| Hypo | 1 (0.82%) | 3 (4.69%) | 0 (0.00%) | 0 (0.00%) | 0 (0.00%) |
| Missing | 51 (41.80%) | 27 (42.19%) | 2 (100.00%) | 2 (33.33%) | 6 (60.00%) |
| **EUS pattern******* |  |  |  |  |  |
| Hypo | 109 (89.34%) | 56 (87.50%) | 1 (50.00%) | 6 (100.00%) | 7 (70.00%) |
| Hyper | 4 (3.28%) | 5 (7.81%) | 0 (0.00%) | 0 (0.00%) | 0 (0.00%) |
| Isoec | 6 (4.92%) | 2 (3.12%) | 1 (50.00%) | 0 (0.00%) | 0 (0.00%) |
| Missing | 3 (2.46%) | 1 (1.56%) | 0 (0.00%) | 0 (0.00%) | 3 (30.00%) |
| **Homogenicity^+^** |  |  |  |  |  |
| Y | 42 (34.43%) | 34 (53.12%) | 0 (0.00%) | 0 (0.00%) | 3 (30.00%) |
| N | 75 (61.48%) | 28 (43.75%) | 2 (100.00%) | 4 (66.67%) | 4 (40.00%) |
| Missing | 5 (4.10%) | 2 (3.12%) | 0 (0.00%) | 2 (33.33%) | 3 (30.00%) |
| **Endoscopist suspic^++^** |  |  |  |  |  |
| Benign | 5 (4.10%) | 19 (29.69%) | 0 (0.00%) | 0 (0.00%) | 1 (10.00%) |
| Malignant | 105 (86.07%) | 22 (34.38%) | 2 (100.00%) | 6 (100.00%) | 6 (60.00%) |
| Undeterminade | 11 (9.02%) | 23 (35.94%) | 0 (0.00%) | 0 (0.00%) | 3 (30.00%) |
| Missing | 1 (0.82%) | 0 (0.00%) | 0 (0.00%) | 0 (0.00%) | 0 (0.00%) |
| **Adrenal morphol^+++^** |  |  |  |  |  |
| “Seagull” | 0 (0.00%) | 2 (3.12%) | 0 (0.00%) | 0 (0.00%) | 1 (10.00%) |
| “Seagull” lateral | 46 (37.70%) | 32 (50.00%) | 0 (0.00%) | 1 (16.67%) | 5 (50.00%) |
| “Seagull” global | 28 (22.95%) | 16 (25.00%) | 0 (0.00%) | 1 (16.67%) | 1 (10.00%) |
| Tumoral | 48 (39.34%) | 11 (17.19%) | 2 (100.00%) | 4 (66.67%) | 3 (30.00%) |
| Others | 0 (0.00%) | 3 (4.69%) | 0 (0.00%) | 0 (0.00%) | 0 (0.00%) |

*Chi2= 7.46, df = 1, p-value = 0.0063 (Malignitat/ Benignitat)

** Chi2= 0.73, df = 1, p-value = 0.3928 ( Malignitat/ Benignitat)

*** Chi2= 5. 50, df = 1, p-value = 0.0190 ( Malignitat/ Benignitat)

**** Chi2= 16.47, df = 1, p-value = 0.000049 ( Malignitat/ Benignitat vs Hypo / Hypo - Normocaptant)

***** Chi2= 0.1214, df = 1, p-value = 0.7275 ( Malignitat/ Benignitat vs Hypoecoica /Hiperecoica-Isoecoica)

+Chi2= 5.20, df = 1, p-value = 0.02257 ( Malignitat/ Benignitat)

^++^Chi2= 54.23, df = 2, p-value <0.00001 ( Malignitat/ Benignitat)

^+++^Chi2= 8.03, df = 2, p-value = 0.01801 ( Malignitat/ Benignitat vs Lateral/Global/tumoral)

| Table 7. Factors related to cytology. | | | | | |
| --- | --- | --- | --- | --- | --- |
| **Variables** | Malignant **No. 122** | Benigne **No. 64** | Atypical cel **No. 2** | Suspicious **No. 6** | Non diag **No. 10** |
| **Needle type*** |  |  |  |  |  |
| Cytological | 92 (75.41%) | 48 (75.00%) | 1 (50.00%) | 3 (50.00%) | 9 (90.00%) |
| Cytohistological | 20 (16.39%) | 9 (14.06%) | 0 (0.00%) | 2 (33.33%) | 0 (0.00%) |
| Missing | 92 (75.41%) | 48 (75.00%) | 1 (50.00%) | 3 (50.00%) | 9 (90.00%) |
| **Needle size**** |  |  |  |  |  |
| 19-gauge | 4 (3.28%) | 2 (3.12%) | 1 (50.00%) | 0 (0.00%) | 0 (0.00%) |
| 20-gauge | 78 (63.93%) | 47 (73.44%) | 0 (0.00%) | 1 (16.67%) | 6 (60.00%) |
| 22-gauge | 37 (30.33%) | 13 (20.31%) | 1 (50.00%) | 3 (50.00%) | 3 (30.00%) |
| 25-gauge | 0 (0.00%) | 2 (3.12%) | 0 (0.00%) | 1 (16.67%) | 0 (0.00%) |
| Missing | 3 (2.46%) | 0 (0.00%) | 0 (0.00%) | 1 (16.67%) | 1 (10.00%) |
| **Technique***** |  |  |  |  |  |
| FNA | 76 (62.30%) | 46 (71.88%) | 1 (50.00%) | 6 (100.00%) | 6 (60.00%) |
| FNA w syringe | 38 (31.15%) | 14 (21.88%) | 1 (50.00%) | 0 (0.00%) | 0 (0.00%) |
| Altres | 3 (2.46%) | 0 (0.00%) | 0 (0.00%) | 0 (0.00%) | 3 (30.00%) |
| Missing | 5 (4.10%) | 4 (6.25%) | 0 (0.00%) | 0 (0.00%) | 1 (10.00%) |
| **Nº of passes****** |  |  |  |  |  |
| Average (DE) | 2.23 (1.31) | 2.15 (1.55) | 4.50 (2.12) | 1.67 (0.82) | 1.60 (0.97) |
| Med (RIQ) | 2.00 (1.00 - 3.00) | 2.00 (1.00 - 3.00) | 4.50 (3.75 - 5.25) | 1.50 (1.00 - 2.00) | 1.00 (1.00 - 2.00) |
| Missing | 3 (2.46%) | 2 (3.12%) | 0 (0%) | 0 (0%) | 0 (0%) |

*Chi2= 0.01471, df = 1, p-value = 0.9035 ( Malignitat/ Benignitat)

**Chi2= 1.6492, df = 1, p-value = 0.1991 ( Malignitat/ Benignitat vs 20/22)

***Chi2= 1.4292, df = 1, p-value = 0.2319 ( Malignitat/ Benignitat)

****W = 3462.5, p-value = 0.3948 ( Malignitat/ Benignitat)

## Objective 2: Evolution

| Taula 8. Factors. | | |
| --- | --- | --- |
| **Variables** | **alive No. 69** | **Exitus No. 101** |
| **Age^** | | |
| Average (DE) | 64.09 (10.01) | 66.39 (9.09) |
| Med (RIC) | 65 (58-70) | 67 (60-73) |
| **Sex¨** | | |
| Man | 46 (66.67%) | 83 (82.18%) |
| Female | 23 (33.33%) | 18 (17.82%) |
| **TC*** | | |
| Normal | 15 (21.74%) | 20 (19.80%) |
| Pathol | 50 (72.46%) | 72 (71.29%) |
| Missing | 4 (5.80%) | 9 (8.91%) |
| **ANR**** | | |
| Y | 49 (71.01%) | 60 (59.41%) |
| N | 19 (27.54%) | 41 (40.59%) |
| Missing | 1 (1.45%) | 0 (0.00%) |
| **≥30***** | | |
| Y | 22 (31.88%) | 53 (52.48%) |
| N | 39 (56.52%) | 40 (39.60%) |
| Missing | 8 (11.59%) | 8 (7.92%) |
| **PET****** | | |
| Hyper | 35 (50.72%) | 48 (47.52%) |
| Normo | 7 (10.14%) | 8 (7.92%) |
| Hypo | 1 (1.45%) | 2 (1.98%) |
| Missing | 26 (37.68%) | 43 (42.57%) |
| **EUS pattern^+^** | | |
| Hypo | 60 (86.96%) | 91 (90.10%) |
| Hyper | 2 (2.90%) | 4 (3.96%) |
| Isoec | 4 (5.80%) | 3 (2.97%) |
| Missing | 3 (4.35%) | 3 (2.97%) |
| **Homogenicity^++^** | | |
| Y | 23 (33.33%) | 43 (42.57%) |
| N | 40 (57.97%) | 53 (52.48%) |
| Missing | 6 (8.70%) | 5 (4.95%) |
| **Suspicious^+++^** | | |
| Benigne | 11 (15.94%) | 7 (6.93%) |
| Malignant | 41 (59.42%) | 76 (75.25%) |
| Undeterminade | 16 (23.19%) | 18 (17.82%) |
| Missing | 1 (1.45%) | 0 (0.00%) |
| **Adrenal morphology^++++^** | | |
| “Seagull” habitual | 1 (1.45%) | 1 (0.99%) |
| “Seagull” lateral | 30 (43.48%) | 42 (41.58%) |
| “Seagull” global | 12 (17.39%) | 21 (20.79%) |
| Tumoral | 24 (34.78%) | 36 (35.64%) |
| Others | 2 (2.90%) | 1 (0.99%) |

^Test t: t = -1.5257, df = 136.7, p-value = 0.1294

¨Chi2= 5.5753, df = 1, p-value = 0.03244

*Chi2= 0.0015543, df = 1, p-value = 0.9686

**Chi2= 2.5578, df = 1, p-value = 0.1097

***Chi2= 5.3282, df = 1, p-value = 0.02098

****Chi2= 0.014471, df = 1, p-value = 0.9042

^+^NA

^++^Chi2= 0.893, df = 1, p-value = 0.3447

^+++^Chi2= 5.4058, df = 2, p-value = 0.06701

^++++^Chi2= 0.21477, df = 2, p-value = 0.8982 (Lateral/Global/Abigarrada)

Table 9. Multivariate logistics model with poor prognosis in evolution (n=106 events 67).

OR IC95% z value Pr(>|z|)

Constant 4.65 1.09 20.99 2.055 0.03989

Age (quinquenis) 1.25 0.99 1.61 1.835 0.06647

Female 0.36 0.13 0.96 -2.026 0.04279

Homogenicity 0.20 0.06 0.56 -2.872 0.00408

Suspicious of malignancy 5.86 1.94 21.00 2.952 0.00316

Figure 1. Multivariate logistics model with poor prognosis in evolution

Area under the curve: 0.75

## Objective 3: Clinical impact

| Taula 10. Related factors. | | |
| --- | --- | --- |
| **Variables** | **Change Y**  **No. 133** | **No No. 69** |
| **CT*** | | |
| Normal | 20 (15.04%) | 18 (26.09%) |
| Pathol | 102 (76.69%) | 43 (62.32%) |
| Missing | 11 (8.27%) | 8 (11.59%) |
| **ANR**** | | |
| Y | 93 (69.92%) | 42 (60.87%) |
| N | 39 (29.32%) | 27 (39.13%) |
| Missing | 1 (0.75%) | 0 (0.00%) |
| **≥30***** | | |
| Y | 67 (50.38%) | 25 (36.23%) |
| N | 58 (43.61%) | 36 (52.17%) |
| Missing | 8 (6.02%) | 8 (11.59%) |
| **PET****** | | |
| Hyper | 72 (54.14%) | 23 (33.33%) |
| Normo | 4 (3.01%) | 12 (17.39%) |
| Hypo | 2 (1.50%) | 2 (2.90%) |
| Missing | 55 (41.35%) | 32 (46.38%) |
| **EUS pattern^+^** | | |
| Hypoec | 118 (88.72%) | 59 (85.51%) |
| Hyperec | 7 (5.26%) | 2 (2.90%) |
| Isoec | 5 (3.76%) | 4 (5.80%) |
| Missing | 3 (2.26%) | 4 (5.80%) |
| **Homogenicity^++^** | | |
| Y | 44 (33.08%) | 35 (50.72%) |
| N | 84 (63.16%) | 27 (39.13%) |
| Missing | 5 (3.76%) | 7 (10.14%) |
| **Suspicious^+++^** | | |
| Benigne | 10 (7.52%) | 14 (20.29%) |
| Malignant | 107 (80.45%) | 33 (47.83%) |
| Undeterminade | 15 (11.28%) | 22 (31.88%) |
| Missing | 1 (0.75%) | 0 (0.00%) |
| **Adrenal morphol^++++^** | | |
| “Seagull” normal | 1 (0.75%) | 1 (1.45%) |
| “Seagull” lateral | 57 (42.86%) | 27 (39.13%) |
| “Seagull” global | 28 (21.05%) | 18 (26.09%) |
| Tumoral | 46 (34.59%) | 21 (30.43%) |
| Others | 1 (0.75%) | 2 (2.90%) |

*Chi2= 3.2731, df = 1, p-value = 0.07043

**Chi2= 1.8084, df = 1, p-value = 0.1787

***Chi2= 2.4161, df = 1, p-value = 0.1201

****Chi2= 10.705, df = 1, p-value = 0.001068

+ Chi2=0.022383, df = 1, p-value = 0.8811

++Chi2= 8.0801, df = 1, p-value = 0.004475

+++Chi2= 22.914, df = 2, p-value = 1.057e-05

++++Chi2= 0.7923, df = 2, p-value = 0.6729

Table 11Multivariate logistics model for therapeutic change

(n=106 events 67).

OR IC95% z value Pr(>|z|)

Constant 2.11 0.38 12.26 0.851 0.3949

Morf. Pathological 2.48 0.84 7.51 1.642 0.1006

ANR 0.36 0.13 0.99 -1.943 0.0520

<30 0.33 0.10 1.04 -1.865 0.0622

Hypo/normo capt. 0.20 0.04 0.82 -2.172 0.0298

No homogenicity 2.73 0.98 7.86 1.915 0.0555

Susp. Pathol 3.71 1.24 11.63 2.316 0.0206

Tumoral 0.36 0.10 1.29 -1.544 0.1225

Lateral 0.24 0.06 0.87 -2.095 0.0361

Figura 2. Multivariate logistics model for therapeutic change

Area under the curve: 0.846

## Objective 4: cases of benign profile

There are 92 patients with a benign profile: normal morphology or hipo / normocaptants or hyper / isocoic or suspected of benign / indeterminate

| Taula 12.Resultat de la citologia en els pacients amb perfil benigne. | |
| --- | --- |
| **Cytology** | |
| Malignant | 36 (39.13%) |
| Benign | 51 (55.43%) |
| Atycal cel | 1 (1.09%) |
| Non diagnons | 4 (4.35%) |

39.1% IC95% [29.2% -49.1%] of patients with a benign profile presented a malignancy result in cytology.

## Objective 5: predictive model of malignancy

| Taula 13.Related factors to malignancy | | |  |
| --- | --- | --- | --- |
| **Variables** | **Normal No. 48** | **Malignant No. 99** | **OR IC95%** |
| **Age** | | |  |
| Average (DE) | 67.79 (8.95) | 63.86 (10.29) |  |
| Med (Q1-Q3) | 67.00 (62.75 - 73.25) | 66.00 (56.00 - 71.00) | 0.81 [0.67-0.97] |
| **Adrenal Size** | | |  |
| Average (DE) | 26.27 (12.07) | 34.76 (17.23) |  |
| Med (Q1-Q3) | 23.00 (19.75 - 33.00) | 30.00 (21.50 - 40.00) | 1.24 [1.08-1.45] |
| **Sex** | | |  |
| Man | 29 (60.42%) | 78 (78.79%) | 1 |
| Female | 19 (39.58%) | 21 (21.21%) | 0.41 [0.19-0.87] |
| **Left/Right** | | |  |
| R | 3 (6.25%) | 8 (8.08%) | 1 |
| L | 45 (93.75%) | 91 (91.92%) | 0.76 [0.16-2.76] |
| **CT** | | |  |
| Normal | 16 (33.33%) | 16 (16.16%) | 1 |
| Pathol | 32 (66.67%) | 83 (83.84%) | 2.59 [1.16-5.84] |
| **AG EUS size** | | |  |
| ≥30 | 16 (33.33%) | 58 (58.59%) | 1 |
| <30 | 32 (66.67%) | 41 (41.41%) | 0.35 [0.17-0.72] |
| **PET** | | |  |
| Hyper | 36 (75.00%) | 96 (96.97%) | 1 |
| Normo/Hypo | 12 (25.00%) | 3 (3.03%) | 0.09 [0.02-0.32] |
| **EUS pattern** | | |  |
| Hypo | 43 (89.58%) | 90 (90.91%) | 1 |
| Hyper/Iso | 5 (10.42%) | 9 (9.09%) | 0.86 [0.28-2.94] |
| **Homogenicity** | | |  |
| Y | 32 (66.67%) | 38 (38.38%) | 1 |
| N | 16 (33.33%) | 61 (61.62%) | 3.21 [1.58-6.75] |
| **Adrenal morphol** | | |  |
| Seagull Lateral | 25 (52.08%) | 36 (36.36%) | 1 |
| seagull Global | 14 (29.17%) | 24 (24.24%) | 1.19 [0.52-2.78] |
| Tumoral | 9 (18.75%) | 39 (39.39%) | 3.01 [1.27-7.62] |

These ORs represent the bivariate association, that is, the factor with the response variable (age vs. malignancy). The interpretation is as follows: For every 5 years less than the average age (65 years) the odds of malignancy increases by 23%. For every 5 mm of more respect the average length (32 mm) the odds of malignancy increases by 24%. Men have an odds of malignancy 2.4 times more than women. Laterality is not associated with malignancy because IC95% includes 1. Glands with a morphology for TAC that are evaluable as pathological have an odds of malignancy 2.6 times greater than non-pathological diseases. Glands with a length ≥30 mm have an odds of malignancy 2.8 times greater than <30mm. The hypercapital glands present an odds of malignancy 11 times greater than hipo / normocaptants. Ecumeicity is not associated with malignancy because the IC95% includes 1. The heterogeneous glands present an odds of malignancy 3.2 times greater than the homogeneous ones. Glands with a global seagull morphology do not present a different odds of malignancy than the glands with a lateral seagull morphology, but the motions that presess odds of malignancy 3 times greater than those of the lateral seagull.

[The word odds can be read as an advantage but not as a risk because we would be overestimated by the magnitude of the association]

Table 14. Logistic model multivariate malignant result

(n=147 events 99).

OR IC95% z value Pr(>|z|)

Constant 1.13 0.50 2.45 0.323 0.74670

Female 0.33 0.13 0.95 -2.537 0.01119

Edat (quinquenis) 0.73 0.56 0.93 -2.731 0.00631

Hetero 3.47 1.34 8.21 3.031 0.00244

AG* Global 1.36 0.55 3.48 0.639 0.52250

AG* tumoral 3.27 1.09 9.61 2.381 0.01725

* Seagull Lateral

Figure 3. Logistic model multivariate malignant result

Sex, age, homogeneity for USE and morphology for USE prove to be the best predictors of a malignant result in a multivariate logistic model. Among the discarded factors are ecogenicity and the size of the gland (<30 vs. ≥30mm). The morphology for TAC, capture by PET, or laterality has not been included as model candidates.

There is a basal gland profile that corresponds to that of a 65-year-old man with a homogeneous gland-like lateral gland. Based on this profile for every 5 years less the odds of malignancy increases by 37%, women have an odds of malignancy 3 times lower, a 3.5 times higher heterogeneous gland and a shrinking 3.3 times larger. However, the interpretation of this OR is not so relevant in this one since the objective of the model is the prediction of malignancy and therefore its properties must be evaluated.

Figure 4. Area under the ROC curve: logistic model multivariate malignant result.

Estimator of the area under the curve:0.78 IC95%[0.70-0.86]

Table 15. Observed expected by the model

|  | Malignant prob | No | Malignant |
| --- | --- | --- | --- |
| D1 | [0.122,0.397] | 11 | 4 |
| D2 | (0.397,0.486] | 9 | 7 |
| D3 | (0.486,0.531] | 5 | 9 |
| D4 | (0.531,0.632] | 8 | 6 |
| D5 | (0.632,0.692] | 6 | 9 |
| D6 | (0.692,0.778] | 3 | 11 |
| D7 | (0.778,0.827] | 2 | 14 |
| D8 | (0.827,0.875] | 1 | 12 |
| D9 | (0.875,0.917] | 2 | 13 |
| D10 | (0.917,0.985] | 11 | 4 |

Depending on the results of table 15, an arbitrary cutting point is set at 0.40. That is, if the probability of malignancy is> 0.40, we would consider the gland as pathological

|  | Pathol | No |
| --- | --- | --- |
| Positive (p≥0,4) | 93 | 29 |
| Negative (p<0,4) | 6 | 19 |

**Estadístics + IC95%**

Sensitivity 93.94% IC95% 87.27% - 97.74%

Specificity 39.58% IC95% 25.77% - 54.73%

Positive Likelihood Ratio 1.55 IC95% 1.23 - 1.97

Negative Likelihood Ratio 0.15 IC95% 0.07 - 0.36

Prevalence 67.35% IC95% 59.13% - 74.85%

Positive Predictive Value 76.23% IC95% 67.68% - 83.47%

Negative Predictive Value 76.00% IC95% 54.87% - 90.64%

## The model has a very good sensitivity (> 90%) but a low specificity (<40%). Therefore, the probability of predicting the positive pathological glands is very high, but the probability of predicting how negative the non-pathological glands is low is low. It is necessary to keep in mind but for a diagnosis confirmation bias, estimation of sensitivity and specificity is very likely to be estimated above and below respectively.

##

## On the other hand, both the probability of having a pathological gland and presenting a positive result as well as the probability of having a non-pathological gland and presenting a negative result is> 75%. Attention but these results are determined by the prevalence. If the estimated prevalence (63%) does not reflect the prevalence of pathological glands observed in these studies, the positive predictive value and the negative predictive value are not credible and should be discarded.

## Annex:

Slide for statistical analysis:

The demographic and clinical characteristics of the patients have been analyzed based on the type of variable and presented in tables.

The predictive model of malignancy of the glands has been constructed through a logistic regression model. The selection process of variables has been automatic from a set of clinically relevant variables (Age, sex, size of the gland by use [<30, ≥30mm], echogenicity [Hypo, Hyper / Iso], homogeneity for USE [EUS, No] and morphology for EUS [global gull, lateral gull, mottled].

The predictive capacity of the model has been evaluated using the area under the ROC curve, the Hosmer-Lemeshow test, the sensitivity / specificity and the positive / negative predictive value.

The program R version 3.1.5 has been used to perform the analysis and treatment of the data.

**Predictive model:**

*Of the factors analyzed at an older age, a pathological morphology by TAC, a length ≥ 30 mm, a hypercapital result by PET, the heterogeneity of the gland by USE and a mottled form by USE presented a risk association with the diagnosis of malignancy. Being a woman turned out to be a protective factor with the diagnosis of malignancy. And finally, laterality and eclecticity did not present any association with the diagnosis of malignancy of the gland.*

*The characteristics mediated by TAC or PET were not taken into account for the construction of the predictive model. The final model included two adjustment factors: sex and age; and two defining factors of the gland measured by USE: homogeneity and morphology [global gull, lateral gull, mottled].*

*The model presented, based on the age, the sex of the patient and the homogeneity and morphology of the gland by USE, allows estimating the probability of a diagnosis of malignancy.*

*Fixed an optimal probability cut point at 0.40, the discriminating capacity of the model was good (AUROC 78% IC95% [70% -86%]). Likewise, sensitivity (93.94% IC95% [87.27% -97.74%]) and positive and negative predictive values ​​(76.23% [IC95%*

*67.68%-83.47%] y 76.00% IC95%[54.87%-90.64%] respectivamente). No así la especificidad (39.58% IC95%[25.77%-54.73%]).*
